# Seismic random noise separation and suppression based on improved variational mode decomposition via grey wolf optimization

**DOI:** 10.1371/journal.pone.0330988

**Published:** 2025-09-05

**Authors:** Zhenjing Yao, Wenzhe Li, Jingyi Zhu, Lei Hao, Mengtao Xing

**Affiliations:** 1 College of Information and Control Engineering, Institute of Disaster Prevention, Sanhe, Hebei, China; 2 Hebei Key Laboratory of Seismic Disaster Instrument and Monitoring Technology, Sanhe, Hebei, China; 3 Langfang Key Laboratory of Accurately Controlled Active Seismic Source, Sanhe, Hebei, China; 4 School of International Education, Beijing University of Chemical Technology, Beijing, China; University 20 Aout 1955 skikda, Algeria, ALGERIA

## Abstract

Seismic noise separation and suppression is an important topic in seismic signal processing to improve the quality of seismic data recorded at monitoring stations. We propose a novel seismic random noise suppression method based on enhanced variational mode decomposition (VMD) with grey wolf optimization (GWO) algorithm, which applies the envelope entropy to evaluate the wolf individual fitness, determine the grey wolf hierarchy, and obtain the optimized key elements *K* and *α* in VMD. Then, the decomposed effective intrinsic mode functions (IMFs) are extracted to separate and suppress random noises. It is worth to be noted that the Kurtosis comparison method can select the IMFs ensuring to preserve valid seismic signal. Finally, the denoised seismic signal is restored by the effective IMFs. The experimental results from synthetic and real field seismic data show that compared with several denoising methods, the proposed method can obtain higher signal-to-noise ratio (SNR) with increasement of 27.78% and lower root mean square error (RMSE) with improvements of 78.82% under the same level of structural similarity (SSIM) which prove the validity and effectiveness of the GWO-VMD method for both separating random noise and preserving valid seismic signal.

## 1. Introduction

The seismic data collected from the monitoring station commonly has random noise, resulting in a reduction of the signal-to-noise ratio for seismic signals. Both before and after earthquakes, the attenuation of random noise in seismic signal is crucial in the seismic data analysis, as it improves the integrity of seismic event [1–3].

In recent decades, there are many seismic noise suppression methods have been proposed, including *t*-*x* domain and transform domain methods [4]. The previous method uses the disparities between noise and signal in the *t-x* domain, characterized by travel time and velocity, such as the polynomial fitting [5], nonlocal means filtering [6] and median filtering [7]. These methods have difficulty accurately distinguishing between noise and effective signals for complex high-density random noise, leading to poor separation performance. The latter method involves converting the seismic signal from the time domain to an alternative domain, where a thresholding operation is then utilized to distinguish and remove noise from signal, such as *f-x* predictive filtering [8], curvelet transform [9], seislet transform [10], S-transform [11,12], Fourier transform [13–15], wavelet transform [16,17]. These methods demonstrate effective results in suppressing noise, but it cannot preserve valid seismic information very well. Especially, these transform domain methods are restricted in terms of time-frequency resolution due to their fundamental decomposition basis. Deep learning-based denoising methods have garnered widespread attention in data processing. These methods typically require a large amount of high-quality seismic data for training to ensure the reliability of the model. However, when faced with small-sample datasets, the model cannot effectively learn denoising knowledge, leading to a reduction in the denoising performance of deep learning-based methods [18–20].

Empirical mode decomposition (EMD) [21] has higher time-frequency resolution which has been successfully applied in seismic noise suppression [22,23]. However, it has the limitations of mode mixing and reactivity to noise which makes it difficult to accurately separate noise from the effective signal. Symmetric geometric mode decomposition (SGMD) [24] effectively preserves fundamental time-series characteristics and demonstrates strong noise robustness. While it struggles with mode decomposition inaccuracies and parameter selection challenges. Variational mode decomposition (VMD) [25] offers a non-iterative strategy for signal decomposition that is both adaptive and nearly orthogonal, thereby enabling it to be an effective method for seismic noise reduction. Xue and colleagues demonstrated a higher spectral and spatial resolution of VMD-based method for seismic time–frequency analysis [26]. Liu *et al*. proposed a denoising method based on VMD for seismic signal, which illustrated the superior performance in both random noise suppression and seismic information extraction [27]. Feng and colleagues proposed a denoising method for seismic signals that combines VMD with a low-rank tensor minimization approach. This method involves decomposing the seismic data in the frequency domain and then forming a seismic tensor to emphasize the frequency characteristics within the seismic data [28]. Zhang *et al*. proposed a multi-channel scheme, referred to as multi-channel variational mode decomposition (MVMD) based on multi-channel singular spectrum analysis (MSSA), for effectively separating and attenuating seismic random noises [29]. Liu *et al*. used multi-channel VMD for identifying the primary components of ground roll, followed by the application of a curvelet-based block-coordinate relaxation method to separate ground roll from reflections [30]. Although the VMD-based techniques are extensively used in the attenuation of seismic random noise, the key parameters of decomposition number *K* and penalty factor *α*, will affect the effectiveness of seismic random noise suppression. *K* with an excessively large value results in a single frequency being segmented into multiple modes, while a too small *K* value leads to frequency mixing. Similarly, a high value of penalty factor *α* may cause the incorrect center frequencies. Conversely, a low value of *α* may bring more noise into a single mode. To address these problems of VMD seismic random noise suppression, the optimal selection of the decomposition number *K* and penalty factor *α* poses a significant challenge [31].

To address the problem of manually selecting the critical parameters *K* and *α*, many researchers have dedicated significant efforts to developing optimization algorithms. Zhang *et al*. proposed a scale-space peak statistical analysis method for determining the *K* value. This method can obtain the *K* value based on the scale-space peak histogram for seismic data [32]. Tian *et al*. integrated a contraction operator mapping into the scale-space representation to construct a strategy for determining the number of IMFs [33]. Li *et al*. developed an empirical equation based on the detrended fluctuation analysis for adaptively determining the number of IMFs for seismic signal reconstruction. However, these approaches are focusing on optimization of the decomposition number *K*, thus the efficiency of the decomposition cannot be maximized [34]. Geetha and colleagues utilized the Grasshopper optimization algorithm to refine the VMD approach for the purpose of enhancing seismic random noise reduction [35]. The energy loss coefficient is selected as the target function to search for the best combination parameters *K* and *α*. But this optimization method has a significant computational load and poor efficiency, which undoubtedly complicates and burdens the optimization algorithm. Whale Optimization Algorithm (WOA) [36] relies solely on an upward spiraling strategy and a simplistic predatory model, exhibiting sensitivity to initial parameters and a propensity for premature convergence. Particle Swarm Optimization (PSO) [37] employing linear velocity updates is characterized by significant parameter sensitivity. While exhibiting rapid initial convergence, the algorithm often stagnates prematurely during later iterations. Firefly Algorithm (FA) [38] simulates firefly attraction based on bioluminescence, where movement is governed by light intensity and relative distance. This model exhibits significant parameter dependency and incurs high computational overhead. Grey wolf optimization (GWO) algorithm [39] is a novel meta-heuristic optimization algorithm has significant advantages, such as a simple configuration, fewer demanded operators, a rapid convergence and powerful competitiveness. Current studies illustrate that this algorithm has outstanding performance in optimization applications [40–43]. Zhang *et al.* proposed a magnetotelluric data denoising method using GWO algorithm, which improved the denoising performance and reliability of low frequency magnetotelluric data [31]. Meseret *et al*. proposed an AGC secondary controller for renewable energy power system based on GWO algorithm, which verified the practicability of GWO method in optimizing the controller parameters [44]. Therefore, it can be proved that the GWO algorithm has a wide range of applicability and practical application in data denoising, parameter optimization, engineering applications and so on. These research results suggest that employing the GWO method to optimize the critical parameters of VMD is viable.

In this study, we propose a novel seismic random noise suppression method by using the GWO algorithm to optimize VMD. First, the grey wolf pack hierarchy is established according to each individual’s fitness value, utilizing envelope entropy assessment. And then, the IMFs are obtained by optimizing key elements *K* and *α* of VMD. Moreover, the effective IMFs are extracted by the Kurtosis comparison method to ensure that the denoised signal contains more valid seismic information. Finally, we recombine the effective IMFs to obtain the denoised seismic data. Through the testing of synthetic seismic data and real field seismic data, the experimental results verify the effectiveness of our proposed GWO-VMD method.

The structure of this paper is arranged as follows. Section II introduces the denoising principle of GWO-VMD method, providing a specific workflow. Section III and IV show the application of our suggested method to synthetic and real-field seismic data, respectively. The efficiency of our method is validated by contrasting it with six established denoising methods. Finally, conclusions are drawn in Section V.

## 2. Methodology

### 2.1. VMD algorithm

VMD is an adaptive algorithm that simultaneously subdivides any input signal into a set of intrinsic mode functions (IMFs) with specified finite bandwidth. In order to ascertain the quasi-orthogonality, it is imperative to minimize the cumulative bandwidth. The constrained variational model is constructed as follows in Equation (1).


$$\begin{array}{c} {}*20cmin\\ \left\{uk,\omega k\right\} \end{array}\left\{\sum {\mathrm{\backslash nolimits}}_{k=1}^{K}{\left\|\partial {\mathrm{\backslash nolimits}}_{t}\left[\left(\delta (t)+\frac{j}{\pi t}\right)*u{\mathrm{\backslash nolimits}}_{k}\left(t\right)\right]e^{-j\omega kt}\right\|}_{2}^{2}\right\}s.t.\sum {\mathrm{\backslash nolimits}}_{k=1}^{K}uk\left(t\right)=x\left(t\right)$$ (1)

where *x*(*t*) is the input signal, *u*_*k*_(*t*) is the IMFs mode, *ω*_*k*_ and *K* are the center frequencies and the number of the IMFs, *δ*(*t*) is the unit impulse function.

In pursuit of the optimal solution for the unconstrained optimization problem, the mode components and their corresponding central frequencies are refined iteratively via the alternating direction method of multipliers. The expressions for the updated mode components and central frequencies are delineated as follows in Equations (2) and (3).


$$u{\mathrm{\backslash nolimits}}_{k}^{n+1}\left(\omega \right)=\frac{x(\omega )-\sum_{k=1}^{k-1}u{\mathrm{\backslash nolimits}}_{k}^{n+1}\left(\omega \right)+\sum_{k=k+1}^{K}u{\mathrm{\backslash nolimits}}_{k}^{n}\left(\omega \right)}{1+2\alpha (\omega -\omega {\mathrm{\backslash nolimits}}_{k}^{n}){\mathrm{\backslash nolimits}}^{2}}$$ (2)

$$\omega {\mathrm{\backslash nolimits}}_{k}^{n+1}=\frac{\int_{0}^{\infty }\omega |u_{k}^{n+1}\left(\omega \right){|}^{2}d\omega }{\int_{0}^{\infty }|u_{k}^{n+1}\left(\omega \right){|}^{2}d\omega }$$ (3)

where *x*(*ω*) is the spectrum of the input signal, *u*(*ω*) is the mode spectrum, *n* is the number of iterations.

The Lagrange multiplier is also iteratively updated in accordance with equation (4) to extract the reconstruction until the specified termination criterion (5) is fulfilled.


$$\lambda^{n+1}\left(\omega \right)=\lambda^{n}\left(\omega \right)+\tau \left(x\left(\omega \right)-\sum {\mathrm{\backslash nolimits}}_{k=1}^{K}u_{k}^{n+1}\left(\omega \right)\right)$$ (4)

$$\sum_{k=1}^{K}\frac{\left\|u{\mathrm{\backslash nolimits}}_{k}^{n+1}\left(\omega \right)-u{\mathrm{\backslash nolimits}}_{k}^{n}\left(\omega \right)\right\|{\mathrm{\backslash nolimits}}_{2}^{2}}{\left\|u{\mathrm{\backslash nolimits}}_{k}^{n}\left(\omega \right)\right\|{\mathrm{\backslash nolimits}}_{2}^{2}}<\varepsilon$$ (5)

where *τ* denotes the step size associated with the Lagrange multiplier term, and *ε* represents the convergence parameter.

In the VMD algorithm, an excessively large decomposition number *K* leads to over-segmentation of frequencies, while an insufficient *K* results in frequency aliasing. Conversely, an excessively high penalty factor *α* may cause erroneous center frequencies, whereas a low *α* allows noise to contaminate individual modes. To address these problem of VMD seismic random noise suppression, an optimization method is employed to search for the optimal values of *K* and *α* in VMD.

### 2.2. GWO algorithm

The grey wolf optimization (GWO) algorithm is a bionic intelligent optimization algorithm that achieves optimization by mimicking the social hierarchy and collaborative hunting behavior of grey wolves. In the social hierarchy, the fittest solution, the second and third best solutions are sequentially named alpha, beta, and delta respectively. The remaining candidate solutions are assumed as omega. In simulating the wolf pack hunting activities, the α-wolf, β-wolf, and δ-wolf are leaders guiding the optimization, while the ω-wolves follow these three wolves. Through tracking, encircling and attacking prey, the grey wolves approach the optimal solution.

Wolf packs employ the following formulae (6)-(7) to search for and encircle prey:


$$D=\left|c\cdot X_{1}\left(t_{0}\right)-X\left(t_{0}\right)\right|$$ (6)

$$X\left(t_{0}+1\right)=X_{1}\left(t_{0}\right)-a\cdot D$$ (7)

$$\left\{ \begin{array}{c} a=2\sigma_{c}\cdot r_{1}-\sigma_{c}\\ c=2r_{2} \end{array}\right.$$ (8)

where *D* is the distance between grey wolf and prey; $t_{0}$ is the current number of iterations; $X_{1}$ indicates the location of prey; *X* indicates the location of the grey wolf; ***a*** and ***c*** are random coefficient vectors, respectively; $\sigma_{c}$ is the convergence factor; $r_{1}$, $r_{2}$ are random vectors in the range of [0, 1].

The α, β and δ wolves determine the optimal solution, guide other grey wolf individuals to update their positions for the final attack as follows in Equation (9):


$${\vec{X}}_{i}^{t+1}=\frac{\left({\vec{X}}_{\alpha }^{t}-A\cdot \left|C\cdot {\vec{X}}_{\alpha }^{t}-{\vec{X}}_{i}^{t}\right|\right)+\left({\vec{X}}_{\beta }^{t}-A\cdot \left|C\cdot {\vec{X}}_{\beta }^{t}-{\vec{X}}_{i}^{t}\right|\right)+\left({\vec{X}}_{\delta }^{t}-A\cdot \left|C\cdot {\vec{X}}_{\delta }^{t}-{\vec{X}}_{i}^{t}\right|\right)}{3}$$ (9)

where *t* is current number of iterations, ${\vec{X}}_{\alpha }^{t}$, ${\vec{X}}_{\beta }^{t}$, ${\vec{X}}_{\delta }^{t}$ are the current position of the prey, ${\vec{X}}_{i}^{i+1}$ is update position, *A* is the adjustment parameter, *C* is the iteration coefficient.

This paper proposes the GWO-VMD seismic denoising algorithm, which utilizes the GWO technique to search for the optimal solutions of *K* and *α* within the VMD, thereby effecting a more comprehensive elimination of noise while preserving a greater degree of the pertinent information within the seismic signal.

### 2.3. GWO-VMD algorithm

The VMD algorithm is capable of decomposing a seismic data into a set of distinct components. This decomposition can be mathematically represented as in Equation (10).


$$x\left(t\right)=\sum_{k=1}^{K}uk\left(t\right)$$ (10)

where *x*(*t*) is the recoded seismic data and *u*_*k*_(*t*) is the *k*th decomposition.

In the GWO-VMD method, a fitness function must be determined to evaluate the individuals within the wolf pack comprising the parameters *K* and α. Envelope entropy constitutes an extension of entropy into the domain of signal envelopes. It correlates with the distribution of envelope constituents and can characterize the degree of uncertainty and complexity inherent within the signal. We employ envelope entropy as a fitness function.

The envelope of IMFs resulting from the VMD decomposition are computed. The summation envelope of all IMFs is expressed as the total envelope. Then the probability distribution *P*_*i*_ of the envelope of each IMF to the total envelope of the recorded signal is calculated using Equation (11). After calculating the envelope entropy *H*_*i*_ of the recorded seismic signal using Equation (12), the fitness value *F*_*i*_ of individual is represented as the minimum value of all IMFs decomposition in Equation (13).


$$Pi=\frac{\sqrt{u_{i}^{2}\left(t\right)+{\left(\frac{\partial ui\left(t\right)}{\partial t}\right)}^{2}}}{\sum_{i=1}^{K}\sqrt{u_{i}^{2}\left(t\right)+{\left(\frac{\partial ui\left(t\right)}{\partial t}\right)}^{2}}}$$ (11)

$$Hi=-\sum_{i=1}^{K}Pi\log Pi$$ (12)

$$Fi=\min{\left|Hi\right|}_{i=1}^{K}$$ (13)

As the kurtosis value using to describe the sharpness of the signal distribution shape, representing the frequency of extreme values, thereby distinguishing the effectiveness of seismic information. This paper proposes combination variational mode decomposition with kurtosis values to select the effective IMFs for seismic signal denoising. Calculating the kurtosis values *K*_*i*_ of the IMFs, those with kurtosis values exceeding the threshold are considered as effective signals, whereas signals with kurtosis values that fall below the established threshold are classified as noise. The corresponding formula is as follows in Equations (14)–(16):


$$Ki=\frac{\frac{1}{N-1}\sum_{j=1}^{N}{\left(u_{i}^{j}-\mu i\right)}^{4}}{{\left(\frac{1}{N}\sum_{j=1}^{N}{\left(u_{i}^{j}-\mu i\right)}^{2}\right)}^{2}}$$ (14)

where *N* is number of IMFs points, $u_{i}^{j}$ is *j*th data of *i*th IMFs decomposition, *μ* is the mean value.


$$ui(t)=\left\{ \begin{array}{c} Sj\left(t\right),Ki\geq Kth\\ Nj\left(t\right),Ki<Kth \end{array}\right.$$ (15)

where *S*_*j*_(*t*) is the valid seismic signal, and *N*_*j*_(*t*) is noise.

The reconstruction signal $\hat{x}(t)$ represented as:


$$\hat{x}\;\left(t\right)=\sum_{j=1}^{m}Sj\left(t\right)$$ (16)

where *m* is the number of effective IMFs.

**Fig 1:**
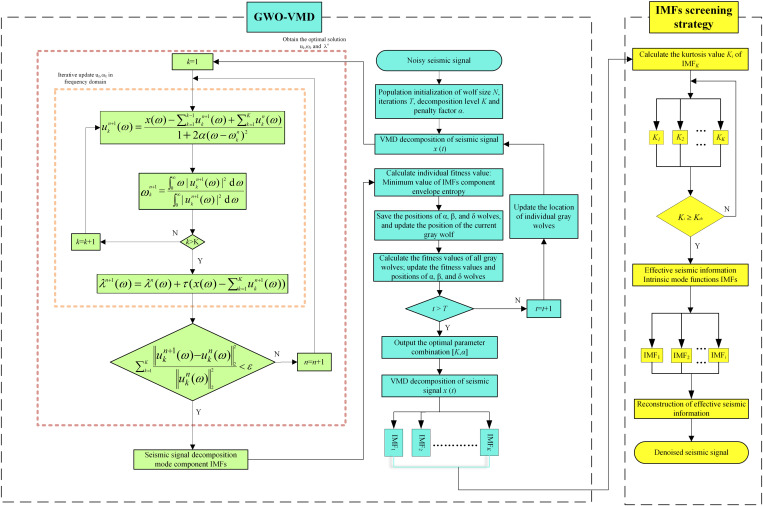
The flowchart of GWO-VMD method.

The flowchart illustrating the proposed GWO-VMD method is depicted in Fig 1. The procedural steps of the GWO-VMD are outlined as follows.

Step 1: The initialization of the population involves setting the parameters for the wolf size *N*, the number of iterations *T*, the decomposition level *K*, and the penalty factor *α*.
Step 2: The seismic signal *x*(*t*) is decomposed to *K* mode component IMFs using VMD.
Step 3: The fitness function is defined as the minimum value of the envelope entropy, which calculates the individual fitness value. Save the best fitness values of the top three wolves as the α, β, and δ wolves, and update the position of the current grey wolf.
Step 4: Calculate the fitness values of all individual wolves and update the positions of α, β, and δ wolves.
Step 5: Iterate steps 2–4 until the predefined maximum number of iterations is attained, and then output the position of the gray wolf with the best fitness value, which is the optimal key parameter combination [*K*, *α*].
Step 6: Decompose the seismic signals using the optimal key parameters within VMD to obtain IMFs components.
Step 7: Calculate the kurtosis value *K*_*i*_ of the IMFs component to separate the effective seismic information from the noise information.
Step 8: Reconstruct the effective seismic component to acquire the denoised seismic signals.

## 3. Synthetic seismic data test

To ascertain the effectiveness of the methodological approach proffered, we have generated synthetic seismic datasets for the purpose of experimentation. To make the simulation analysis closer to the actual seismic reality, the synthetic signal should be closer to the field seismic signal. The synthetic seismic data is generated by Bumps wave and Ricker wavelet with a single-channel, and Ricker wavelet profile of 40 seismic sections. The parameter *K* is typically set within an integer range of [2,10] and *α* is generally established as a continuous value interval of [100, 5000]. The population size of the GWO algorithm usually comprises 10–30 individuals. To ensure search efficiency in low-dimensional optimization problems, the grey wolf size *N* is set to 20. The maximum number of iterations is set between 50 and 200, with the specific value determined by observing convergence curves. We set it to 50. In internal iteration, the convergence tolerance maintains the default value of 1e-6.

To assess the efficacy of noise attenuation, the Signal-to-Noise Ratio (SNR), Root Mean Square Error (RMSE) and Structural Similarity Index (SSIM) are established as the metrics for performance evaluation in Equations (17)–(19).


$$\text{SNR}=20lg\frac{{\sum {\mathrm{\backslash nolimits}}_{i=1}^{N}\left(x\left(i\right)\right)}^{2}}{{\sum {\mathrm{\backslash nolimits}}_{i=1}^{N}\left(\hat{x}\left(i\right)-x\left(i\right)\right)}^{2}}$$ (17)

$$\text{RMSE}=\sqrt{\frac{1}{N}\sum {\mathrm{\backslash nolimits}}_{i=1}^{N}{\left(\hat{x}\left(i\right)-x\left(i\right)\right)}^{2}}$$ (18)

$$\text{SSIM}=\frac{\left(2\mu_{x}\mu_{y}+C_{1}\right)\left(2\sigma_{xy}+C_{2}\right)}{\left(\mu_{x}^{2}+\mu_{y}^{2}+C_{1}\right)\left(\sigma_{x}^{2}+\sigma_{y}^{2}+C_{2}\right)}$$ (19)

where $\mu_{x}$ and $\mu_{y}$ are the means of signals *x* and *y*, $\sigma_{x}$ and $\sigma_{y}$ are the standard deviation of signals *x* and *y*, $\sigma_{xy}$ is the covariance of signals *x* and *y*, and $C_{1}$ and $C_{2}$ are the stability constants.

### 3.1. Bumps signal test

**Fig 2:**
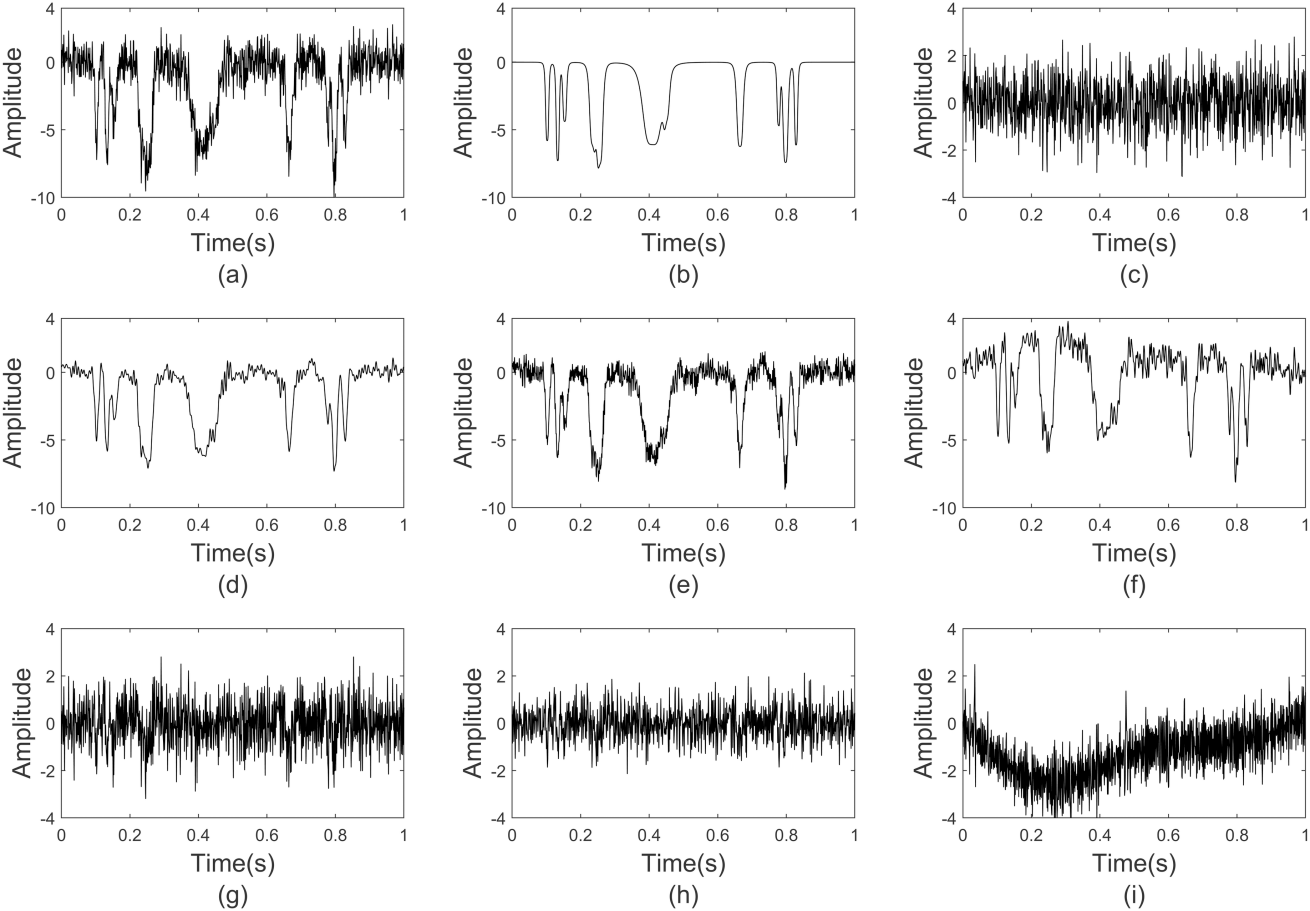
Noise reduction performance comparison on synthetic Bump data. (a) noisy synthetic data, (b) Noise-free synthetic data, (c) random noise, (d) denoising signal waveform based GWO-VMD, (e) denoising signal waveform based VMD, (f) denoising signal waveform based EMD, (g) denoised noise waveform based GWO-VMD, (h) denoised noise waveform based VMD, (i) denoised noise waveform based EMD.

The Bumps signal is a periodic signal with being abrupt features of seismic fields shown in Fig 2a. The sampling interval is 1ms. A Bumps wave with the amplitude 20 and duration time 1s is used to generate the synthetic data in Fig 2b. Then, -5dB random noise in Fig 2c is superimposed onto the synthetic data.

We apply the GWO-VMD, VMD and EMD methods to the noisy synthetic signal and obtain the denoising signal waveform which are displayed in Fig 2d–2f, respectively. Corresponding denoised noise waveform are shown in Fig 2g–2i, respectively. It can be found that the waveform after denoising process from 0.75 s to 0.85 s are obviously deformed using the VMD and EMD methods. However, the waveform is smoother without additional deformations after processing using GWO-VMD method, which illustrates that it is useful for the random noise attenuation and valid signal reservation.

To further test the performance of the GWO-VMD method, grasshopper optimization algorithm VMD (GOA-VMD), discrete wavelet transform (DWT), K-means clustering algorithm (K-means), and convolutional neural network (CNN) denoising methods are added for comparative analysis. Table 1 provides a statistical analysis of the Bump denoising results for the seven methods. The synthetic Bump signal is added random noise, varying in intensity from −20 dB to 10 dB in steps of 5 dB. From Table 1, it is evident that the application of the GWO-VMD method for denoising synthetic Bump signals across varying levels of noise level, the SNR values exhibit an increase. Concurrently, RMSE values demonstrate a reduction, indicative of improved denoising performance. Furthermore, SSIM values are observed to increase, reflecting an improvement in the preservation of the structural information within the denoised signals. So the random noises are successfully removed from the synthetic data and the valid signal are reserved better than the other six methods.

**Table 1:** Comparison of denoising method with synthetic Bump data.

Noise level (dB)	Metrics	Denoising method						
		GWO-VMD	VMD	EMD	GOA-VMD	DWT	K-means	CNN
−20	SNR	−10.70	−15.19	−16.18	−11.70	−19.95	−17.24	−14.61
	RMSE	6.4103	10.7450	12.0408	7.1933	18.5852	13.6156	10.0511
	SSIM	0.8327	0.3966	0.5806	0.7658	0.0093	0.3208	0.5283
−15	SNR	−4.55	−9.81	−10.66	−5.07	−14.54	−12.56	−9.60
	RMSE	3.1575	5.7829	6.3813	3.3534	9.9678	7.9379	5.6470
	SSIM	0.8881	0.4577	0.5918	0.8572	0.0129	0.1104	0.6031
−10	SNR	1.09	−5.67	−5.80	0.67	−9.99	−9.46	−4.73
	RMSE	1.6494	3.5934	3.6450	1.7307	5.9095	5.5588	3.2231
	SSIM	0.9060	0.3465	0.5511	0.8548	0.1798	0.1213	0.7086
−5	SNR	6.15	0.89	−1.27	4.20	−3.99	−3.69	−0.25
	RMSE	0.9214	1.6887	2.1643	1.1535	2.9611	2.8584	1.9235
	SSIM	0.9717	0.8735	0.7312	0.9397	0.5024	0.6234	0.8283
5	SNR	12.44	9.87	3.30	8.84	3.99	1.20	5.40
	RMSE	0.4467	0.6004	1.2796	0.6762	1.1810	1.6286	1.0043
	SSIM	0.9921	0.9895	0.9252	0.9752	0.9596	0.8986	0.9786
10	SNR	17.92	8.77	4.00	10.15	5.91	1.40	6.15
	RMSE	0.1583	0.4536	0.7856	0.3869	0.9467	1.5915	0.9214
	SSIM	0.9986	0.9738	0.9532	0.9775	0.9729	0.9135	0.9894

### 3.2. Single-channel Ricker signal test

**Fig 3:**
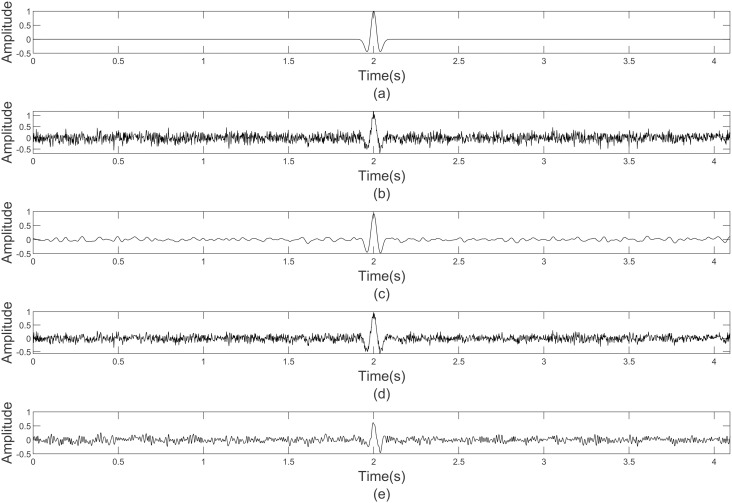
Noise reduction performance comparison on synthetic Ricker data. (a) Noise-free synthetic data, (b) noisy data, (c) denoised data based GWO-VMD, (d) denoised data based VMD, (e) denoised data based EMD.

The Ricker wavelet is the most similar to seismic signal with characteristic of time-varying and non-stationary. Fig 3a displays a single-channel Ricker signal with the dominant frequency 10 Hz and 600 sampling points. Fig 3 demonstrates the synthetic seismic data of Ricker wavelet under the −5 dB noise level. The outcomes of noise reduction for the comparative methods are depicted in Fig 3c–3e. It can be observed from the denoised segment in Fig 3c that compared with VMD and EMD methods, the denoised wave of the proposed GWO-VMD method is smoother and closely matches the noise-free signal. This observation confirms that the proposed GWO-VMD method is capable of effectively eliminating random noise while preserving the pertinent seismic information.

**Fig 4:**
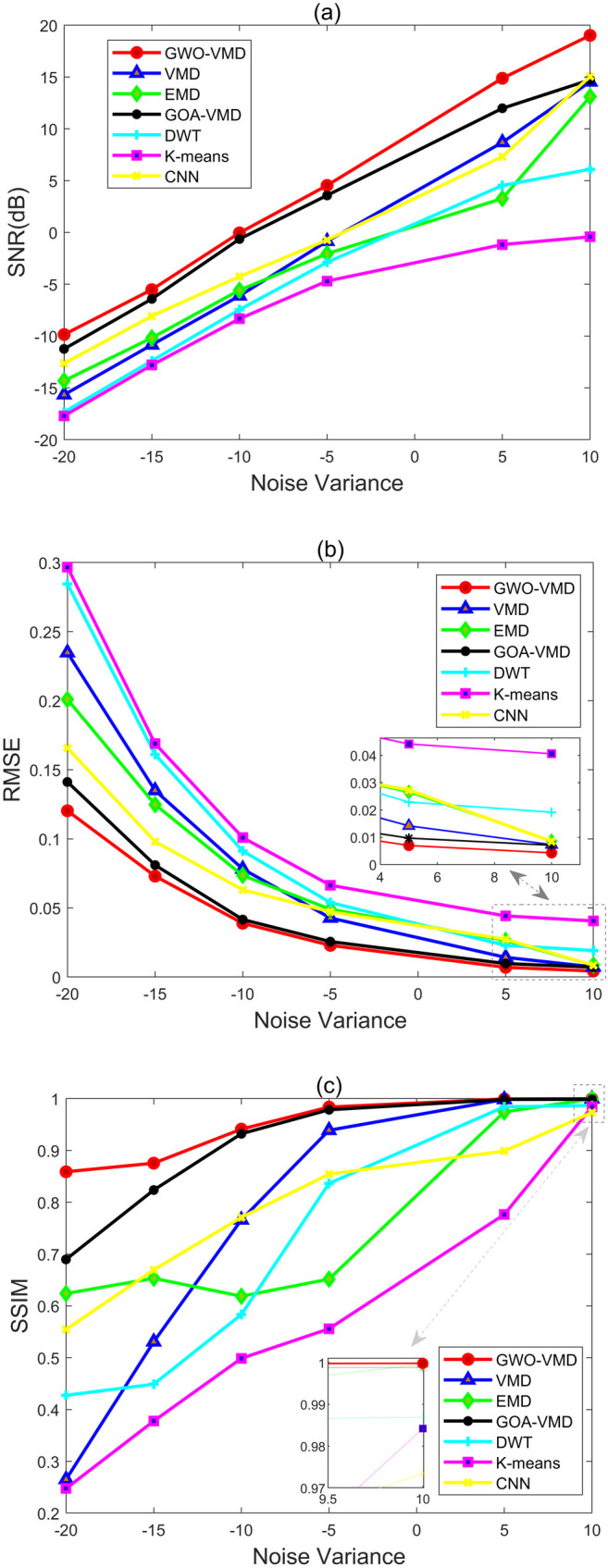
Comparison across various methods at different noise levels. (a) SNR, (b) RMSE, (c) SSIM.

In order to further evaluate the efficacy of the GWO-VMD method, we added random noise with different SNR levels of −20, −15, −10, −5, 0, 5, 10 dB, respectively. As well as the methods being compared are the including thee GWO-VMD, VMD, EMD, GOA-VMD, DWT, K-means and CNN noise reduction technique. The denoising results have been shown in Fig 4, we can see that as the noise intensity decreases, the SNR and SSIM of the three seven comparison methods show an increasing trend and the RMSE illustrates a decreasing trend. So contrasted with above methods, the proposed method has notably enhanced the noise reduction capability.

### 3.3. Synthetic Ricker seismic profile test

**Fig 5:**
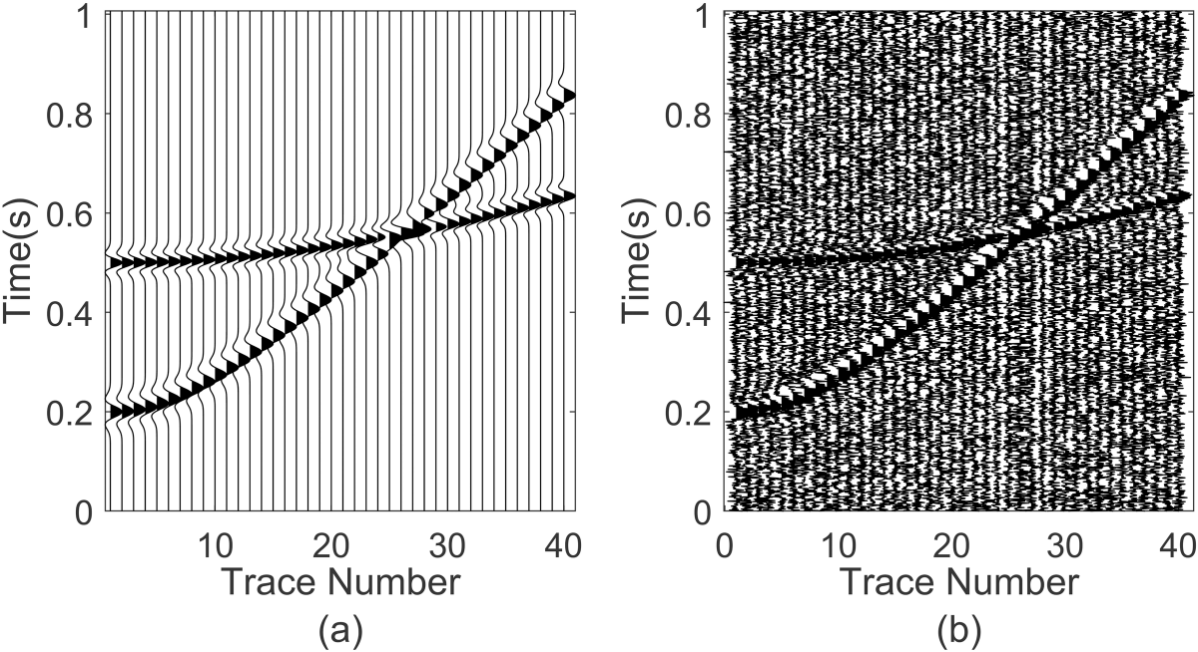
Ricker seismic profile. (a) Noise-free profile, (b) noisy profile.

In this section, the synthetic seismic profile has been used to test the performance of GWO-VMD. Fig 5a shows the noise-free synthetic Ricker profile which has 40 traces and 1000 sampling points in each trace. The dominant frequency is set to 15 Hz with a peck of 1 and a spindle speed of 1200 m/s. The superimposed domain frequency is set to 20 Hz with a peak of 1.5 and a spindle speed of 2500 m/s. Its trace interval distance is set to 30 m. We add −3 dB Gaussian to the synthetic profile, which is presented in Fig 5b.

**Fig 6:**
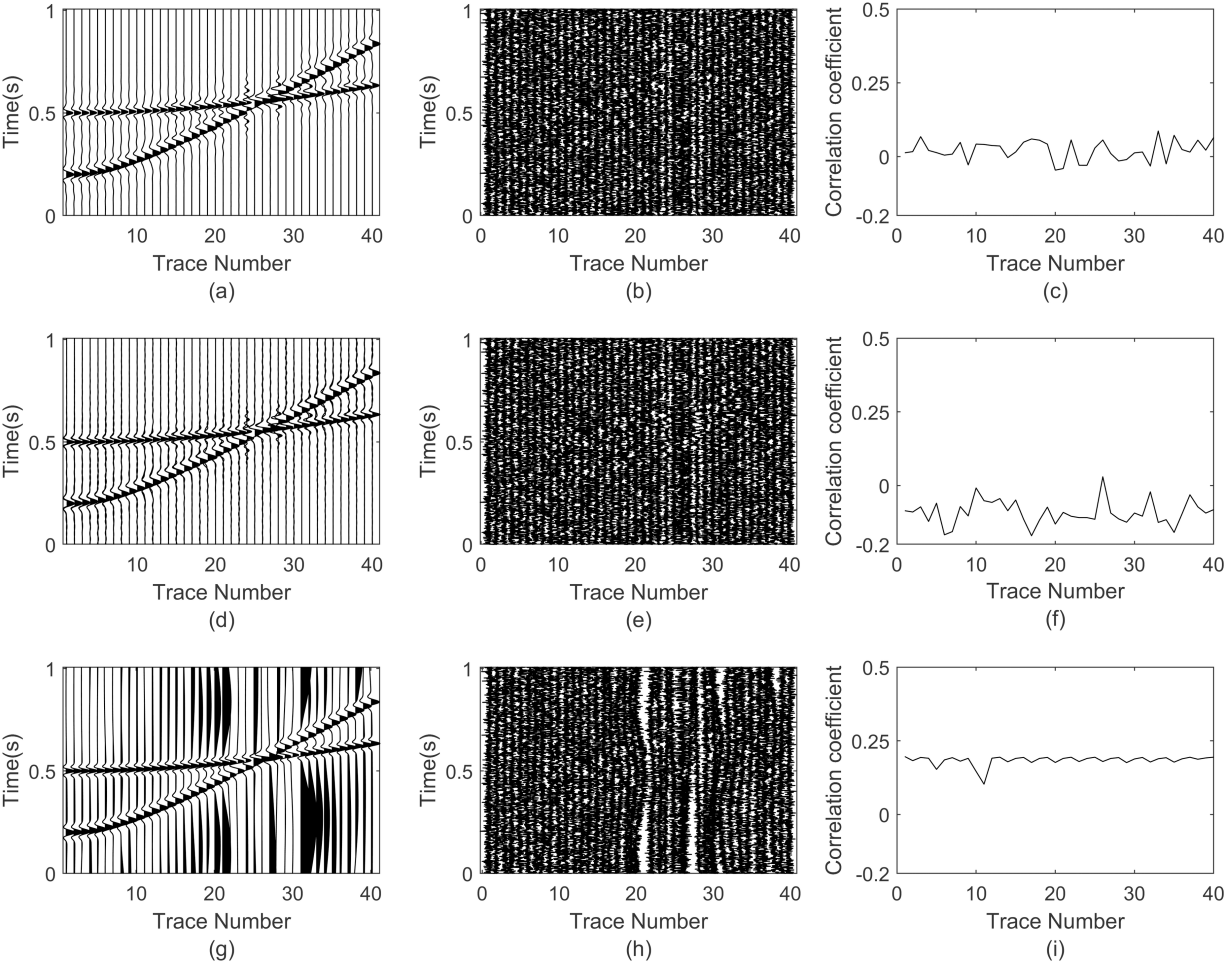
Noise reduction performance comparison on synthetic Ricker profile. (a) denoised synthetic profile based GWO-VMD, (b) removed noise based GWO-VMD, (c) correlation coefficient of (a) and (b), (d) denoised synthetic profile based VMD, (e) removed noise based VMD, (f) correlation coefficient of (d) and (e), (g) denoised synthetic profile based EMD, (h) removed noise based EMD, (i) correlation coefficient of (g) and (h).

The GWO-VMD, VMD and EMD methods are applied to the noisy synthetic Ricker profile and get the denoised profile in Fig 6. The denoised profile with GWO-VMD are well separated the random noise from the seismic events as illustrated in Fig 6a and 6b. While Fig 6d and 6e shows that the denoised profile based the VMD has remove behind a portion of noise. In Fig 6g, denoised profile based the EMD is obviously lost the effective seismic information. The blank space in Fig 6h illustrates that the separated noise is different from the added Gaussian noise. We can calculate the Pearson correlation coefficient between the removed noise and the valid seismic signal, which is useful for testing the performance quantitatively. A lower correlation coefficient indicates a superior noise reduction performance, suggesting that the noise has been effectively mitigated while preserving the integrity of the underlying signal. Comparison of Fig 6c, 6f and 6i, the correlation coefficient of EMD method fluctuates around 0.1, based-VMD is in the vicinity of 0.05, while the majority of coefficient with GWO-VMD is significantly less than 0.05. The results indicate that the proposed GWO-VMD methodology is superior in terms of random noise suppression and the preservation of seismic signal.

Table 2 presents a comparative analysis of the SNR, RMSE, and SSIM for synthetic seismic profile signal after denoising using seven different methods. The statistical data indicate that compared to other six methods, the proposed GWO-VMD significantly enhances the SNR post-denoising, at least increasing from 10.53 dB to 16.73 dB with improvements of 58.87%. Additionally, the RMSE is reduced from 0.0406 to 0.0086 with reduction of 78.82%. The SSIM is also elevated from 0.9867 to 0.9992, indicating a higher degree of structural similarity. Therefore, it can be concluded that the GWO-VMD method exhibits superior performance in random noise attenuation and seismic signal preservation, providing a more effective denoising method.

**Table 2:** Comparison of denoising method with synthetic Ricker seismic profile.

Signal	Metrics	Denoising method						
		GWO- VMD	VMD	EMD	GOA- VMD	DWT	K-means	CNN
**Synthetic Ricker seismic profile**	SNR	16.73	3.26	1.45	10.53	4.62	0.27	8.86
	RMSE	0.0086	0.0406	0.0500	0.1760	0.0776	0.0746	0.0535
	SSIM	0.9992	0.9791	0.9802	0.9867	0.7573	0.5924	0.9244

## 4. Filed seismic data application

In this section, we utilize the GWO-VMD approach to test its performance on real field seismic data. To begin with, we make use of an open global dataset of seismic event records. Then the real field data, which was recorded over the Setouchi city, Japan. Finally, a 2D field seismic section comprising 200 seismic traces employed for the application.

### 4.1. Stanford Earthquake Dataset noise suppression

**Fig 7:**
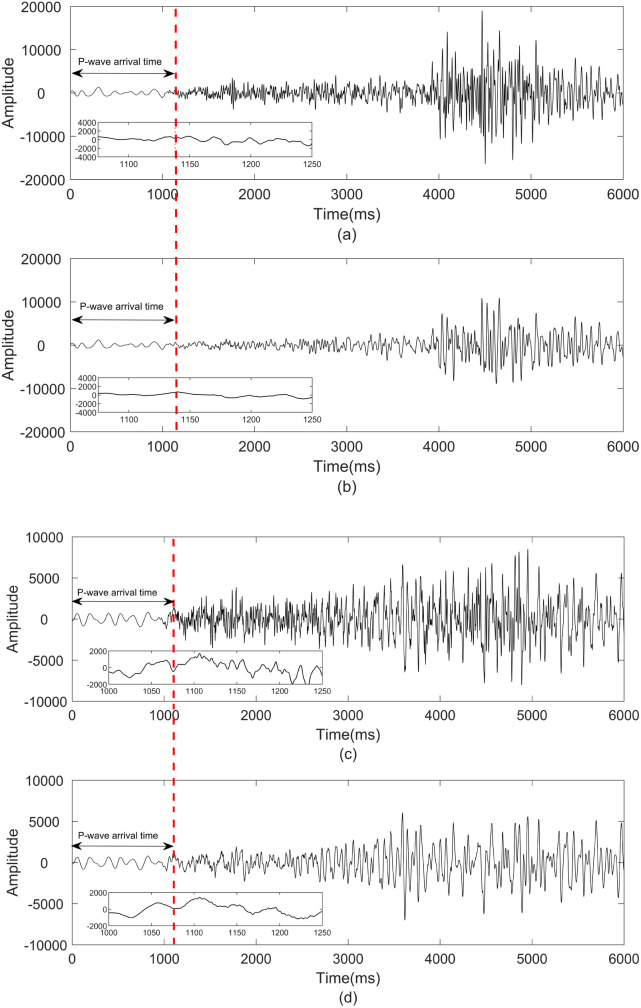
Noise reduction on testing sets from STEAD.

The Stanford Earthquake Dataset (STEAD) is employed for earthquake prediction, which constitutes an open-access global dataset of seismic event records. To further evaluate the performance of the proposed GWO-VMD method, denoising tests were conducted on two different test sets from STEAD dataset. The sampling points is 6000. In Fig 7, the recorded seismic signal in the STEAD dataset and the amplified signal with the P-wave arrival time after noise reduction by the GWO-VMD algorithm are shown. The smoother waves in Fig 7b and 7d clearly indicate the removal of random noise from the seismic signal. Moreover, the P-wave arrival time accurately locate in denoise seismic signal based on GWO-VMD method. It demonstrates that the proposed algorithm can usefully eliminate the noise and preserve the most effective seismic signal, which improves the data quality of the STEAD dataset.

### 4.2. Real field seismic signal noise suppression

**Fig 8:**
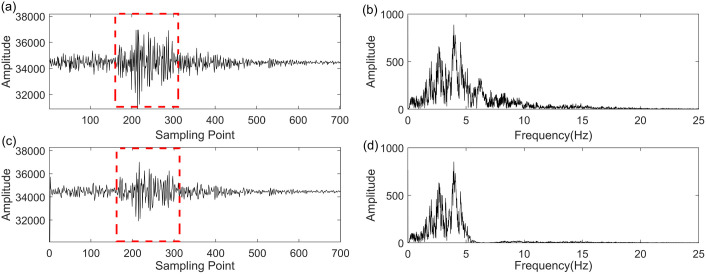
Noise reduction performance on real field seismic data of UD direction at Setouchi city. (a) recorded seismic signal, (b) frequency-domain waveform of (a), (c) denoised seismic signal based GWO-VMD method, (d) frequency-domain waveform of (c).

**Fig 9:**
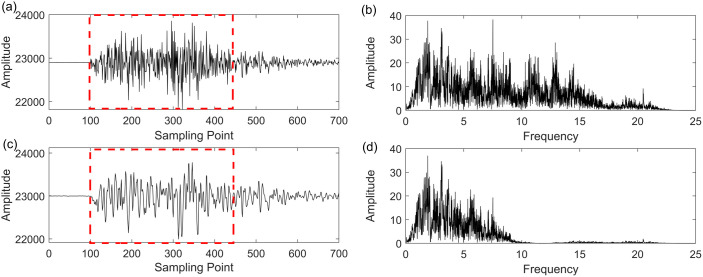
Noise reduction performance on real field seismic data of NS direction at Setouchi city. (a) recorded seismic signal, (b) frequency-domain waveform of (a), (c) denoised seismic signal based GWO-VMD method, (d) frequency-domain waveform of (c).

**Fig 10:**
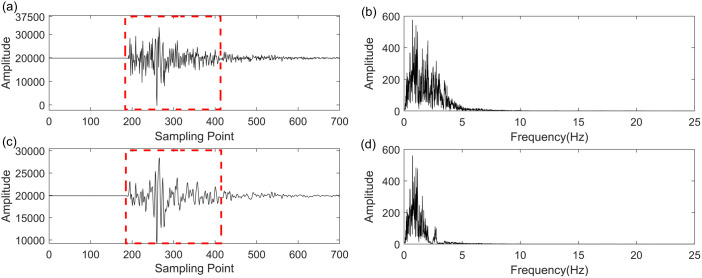
Noise reduction performance on real field seismic data of EW direction at Setouchi city. (a) recorded seismic signal, (b) frequency-domain waveform of (a), (c) denoised seismic signal based GWO-VMD method, (d) frequency-domain waveform of (c).

In this section, we employ the GWO-VMD methodology to analyze real seismic field data, with the objective of assessing its efficacy and utility in a practical seismic signal processing context. The real field seismic signal was acquired in the Setouchi city, Japan. Figs 8–10 illustrate the seismic data, the denoised seismic signal and frequency-domain waveform at Setouchi city in NS, EW and UD direction, respectively. In Figs 8c, 9c and 10c, the denoised seismic signal mainly retained valid seismic information and removed the random noise to a certain extent. Thus, the frequency distribution in Figs 8d, 9d and 10d also demonstrates the proposed method has significant performance improvement in noise reduction especially above 5 Hz. The proposed method in retaining the valid seismic information displayed by the red rectangles. The outcomes illustrate the proficiency of the GWO-VMD approach in diminishing noise within the real field seismic signal, thereby validating its noise reduction capabilities.

### 4.3. Real field seismic profile noise suppression

**Fig 11:**
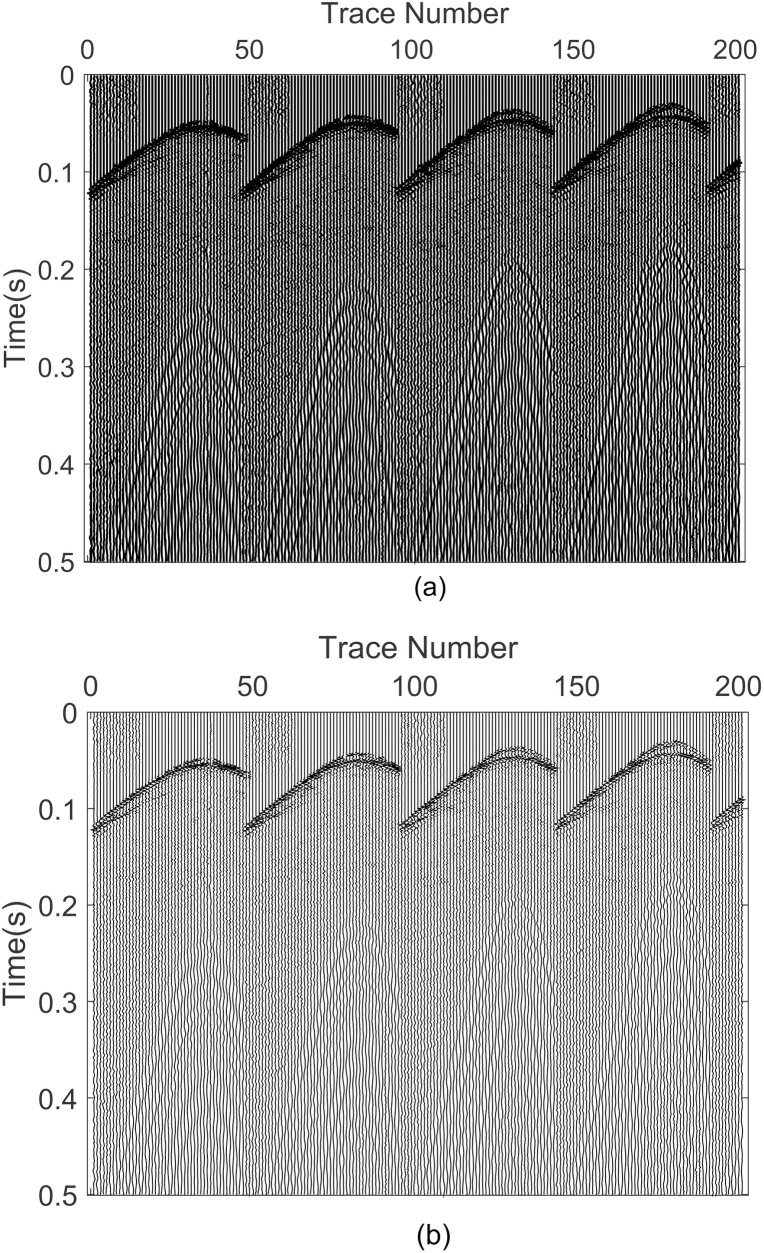
Noise reduction performance of 2D seismic profile based on GWO-VMD. (a) 2D actual seismic data, (b) denoised seismic data.

In this section, a 2D field seismic profile is chosen for the purpose of conducting an additional assessment of the performance characteristics of the aforementioned noise reduction methods. Fig 11a shows the field seismic profile section, which consists of 200 seismic traces, each with 500 sampling points. Fig 11b illustrates the denoising result based on the proposed approach. The comparison between Fig 11a and 11b reveals that the seismic profile has been successfully separated from random noise, enhancing the resolution to a certain extent.

**Fig 12:**
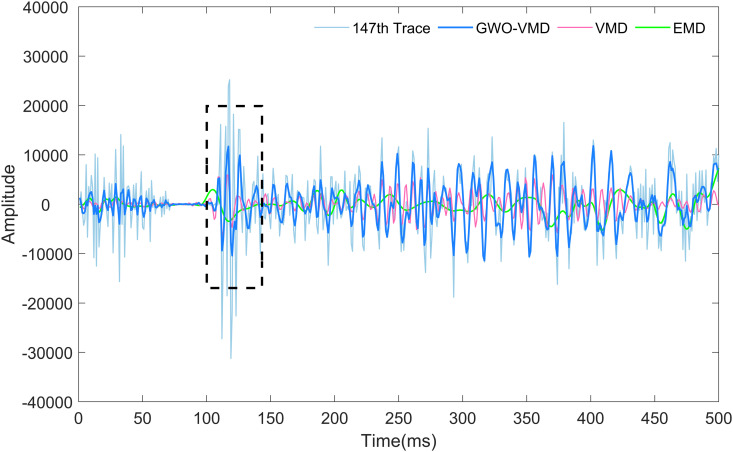
The 147th single trace.

To substantiate the practical utility of the proposed method further, a single seismic trace is extracted and presented in Fig 12 for analysis. It is important to mention that the number of the trace is 147. The grey, blue, pink and green curves illustrate the seismic data, and denoised traces achieved through our method, VMD method, and EMD method, respectively. The suggested approach demonstrates superior performance compared to the other methods in preserving the seismic information highlighted by the black rectangles. This single trace illustration serves to validate and highlight the effectiveness of our proposed method for random noise removal and reduction.

Further increase four methods of GOA-VMD, DWT, K-means and CNN to compare the real field seismic profile denoising effect, the experimental results are shown in Table 3. Table 3 illustrates the statistical comparison of real field seismic profile noise reduction results of the seven methods in terms of SNR. We can see that the proposed GWO-VMD method improves SNR better than other six methods at least 9.97 dB to 12.74 dB with an increase of 27.78%. The outcomes derived from the field profile show that the proposed method successfully removes random noise while maintaining the valid seismic signal.

**Table 3:** SNR comparison of real-field seismic profile.

Seismic data	GWO-VMD	VMD	EMD	GOA-VMD	DWT	K-means	CNN
**200 seismic signals**	12.74	6.07	5.91	6.61	−0.56	3.37	9.97

## 5. Conclusion

This article proposed a novel GWO-VMD denoising method for separating and suppressing seismic random noise based on GWO and VMD. The optimizing critical parameters *K* and *α* are obtained by using envelope entropy as fitness function to evaluate the grey wolf hierarchy. Through Kurtosis comparison method, the effective IMFs are determined to reconstruct seismic signal avoiding ineffectively denoising, which can simultaneously reduce random noises and retain valid signals. The experimental results on synthetic and real field seismic data illustrate the effectiveness of our proposed method separating random noise compared with the EMD and VMD denoising methods. The GWO-VMD algorithm continues to face performance challenges with large-scale data processing, even with the adoption of parallelization and boundary processing optimization strategies. Concretely, while it takes 16 seconds to process 1000 samples, handling 10,000 samples results in a significantly longer processing time of approximately 226 seconds. In the future research, we plan to explore more denoising method with high computational efficiency to suppress different types of seismic noise. Moreover, we have currently only tested the effectiveness of the proposed workflow in separating and attenuating seismic random noise. While seismic data contains various types of coherent noises, such as ground roll, scattered waves, and diffracted waves. We will conduct further research, such as Symmetric geometric mode decomposition, sparse time-frequency analysis, and deep learning methods to separate and attenuate seismic coherent noises.
